# Supplementary material for: Electrically induced cancellation and inversion of piezoelectricity in ferroelectric Hf0.5Zr0.5O2
Source: Nat Commun. 2024 Jan 29;15:860. doi: 10.1038/s41467-024-44690-9 (PMC10825184; doi:10.1038/s41467-024-44690-9)
Supplement: Supplementary file 1 — Supplementary Information [file 41467_2024_44690_MOESM1_ESM.docx]

**Supporting Information**

**Electrically-induced cancellation and inversion of piezoelectricity in ferroelectric Hf_0.5_Zr_0.5_O_2_**

Haidong Lu^1+^, Dong-Jik Kim^2+^, Hugo Aramberri^3^, Marco Holzer^2,4^, Pratyush Buragohain^1^, Sangita Dutta^3,5^, Uwe Schroeder^6^, Veeresh Deshpande^2^, Jorge Íñiguez^3,5*^, Alexei Gruverman^1*^, Catherine Dubourdieu^2,4^*

1. Department of Physics and Astronomy, University of Nebraska-Lincoln, Lincoln, NE 68588-0299, USA

2. Helmholtz-Zentrum Berlin für Materialien und Energie, Insitute Functional Oxides for Energy-Efficient Information Technology, Hahn Meitner Platz 1, 14109 Berlin, Germany

3. Materials Research and Technology Department, Luxembourg Institute of Science and Technology (LIST), Avenue des Hauts-Fourneaux 5, L-4362 Esch/Alzette, Luxembourg

4. Freie Universität Berlin, Physical Chemistry, Arnimallee 22, 14195 Berlin, Germany

5. Department of Physics and Materials Science, University of Luxembourg, Rue du Brill 41, L-4422 Belvaux, Luxembourg

6. NaMLab gGmbH, Noethnitzer Strasse 64 a, 01187 Dresden, Germany

+ these authors contributed equally

* Corresponding authors: [catherine.dubourdieu@helmholtz-berlin.de](mailto:catherine.dubourdieu@helmholtz-berlin.de), [agruverman2@unl.edu](mailto:agruverman2@unl.edu), [jorge.iniguez@list.lu](mailto:jorge.iniguez@list.lu)


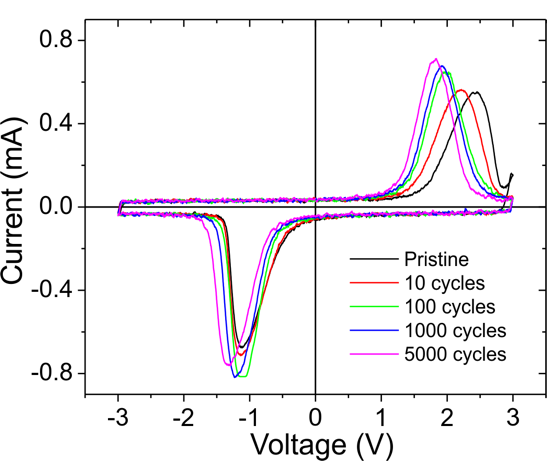


**Fig S1**: I-V curves corresponding to the P-V loops shown in Fig. 1(a).

Figure S2 illustrates polarization distribution and switching in the W/HZO/W capacitor with zero net piezoresponse achieved after ac cycling. The zero net piezoresponse is due to the equal fractions of the regions with positive and negative *d_33_*. The net piezoresponse signal, evaluated by integrating the local piezoresponse over the area shown in Fig. S2(a-d) from a set of PFM images of the capacitor after applying poling pulses of variable amplitudes, is almost zero due to the mutual cancellation of the signals from the regions with positive and negative *d_33_* (Fig. S2(f)). A hysteresis voltage dependence of the negative and positive piezoresponse signals confirms that regions with both positive and negative *d_33_* are perfectly switchable (Fig. S2(g)).


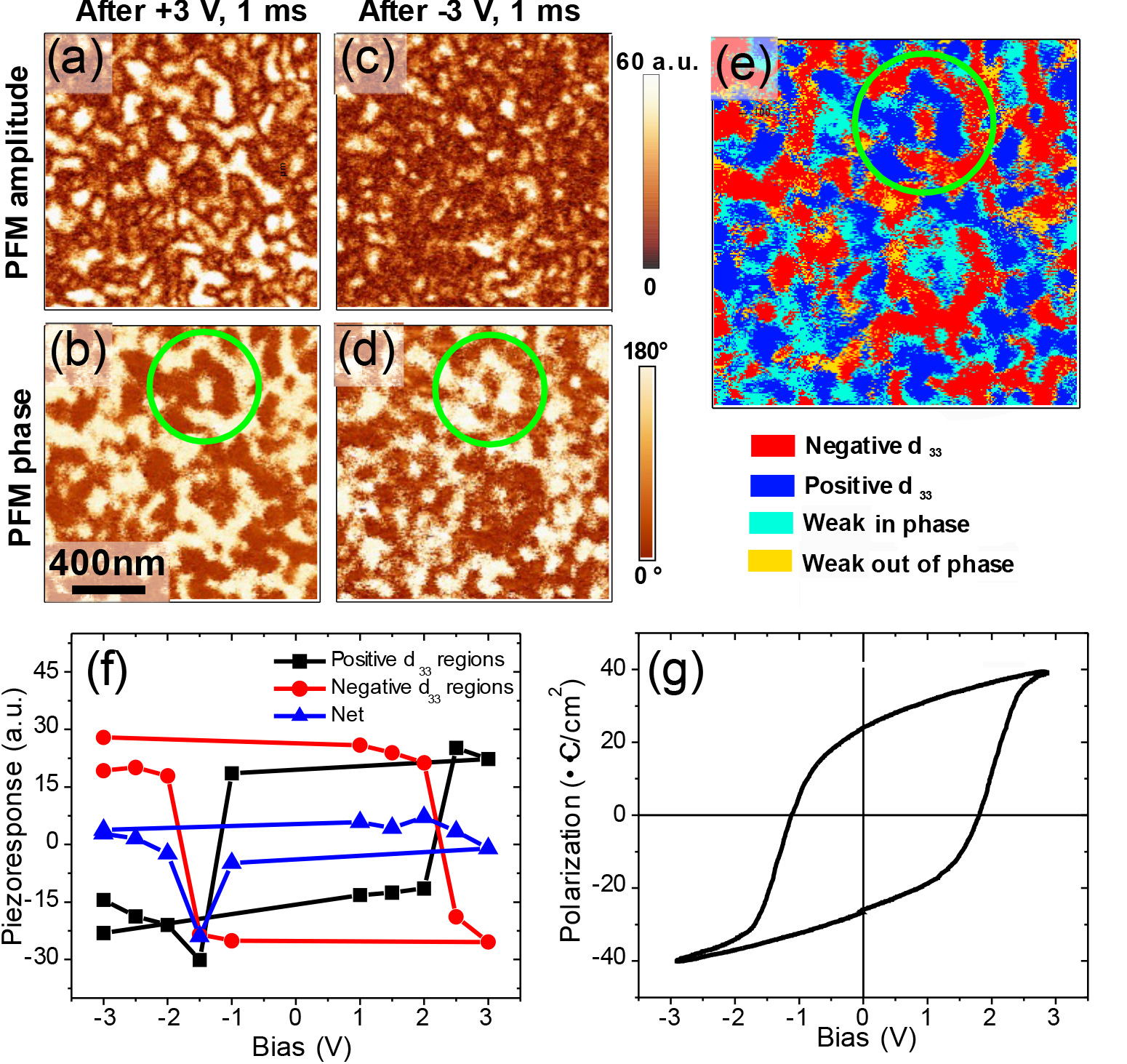


**Fig. S2. Polarization switching in the W/HZO/W capacitor with zero net piezoresponse.** (a-d) PFM amplitude and phase images after + 3 V, 1 ms pulse (a,b), and after - 3 V, 1 ms pulse (c,d), respectively. Green circles in (b) and (d) highlight regions with mutually opposite changes in PFM phase contrast – a signature of opposite *d_33_* in these regions. (e) Regions with positive and negative *d_33_* extracted from (b) and (d). (f) Cumulative piezoresponse signals from the regions with positive and negative *d_33_*, obtained by integrating the local piezoresponse over the area shown in (a-d) from a set of PFM snapshots of the domain patterns produced by a sequence of poling pulses of variable amplitudes (not shown here), and a net piezoresponse signal from the entire region shown in (a-d). (g) The corresponding P-V hysteresis from the capacitor with zero net piezoresponse.

Figure S3 illustrates, on two different W/HZO/W capacitors (named L and R), the gradual evolution of the *d_33_* positive and negative regions with a very progressive electrical cycling. Here we highlight, by local PFM switching spectroscopy (phase and amplitude) on the same fixed location of the PFM images, that the initial positive *d_33_* coefficient at local level gradually decreases, goes through the zero value, and then becomes negative (change of the phase signal) with a progressive increasing amplitude upon further ac cycling. This gradual change of the PFM spectroscopy amplitude and the change of the phase signal as a function of ac training cycles suggest that the inversion of the *d*_33_ coefficient at the local level is not instantaneous but rather gradual and that there is a state where *d_33_* is zero (see Fig. 3g of the manuscript). The hysteresis P-V loops measured for the capacitor after each local switching spectroscopy measurement show a robust ferroelectricity not only when the net *d_33_* of the capacitor is zero (achieved for around 21 cycles in capacitor R as shown by the PFM images of Fig. S3 (C) a, but also throughout the whole ac cycling process, including when the local *d_33_* passes through zero. There is a continuous progressive increase of the remanent polarization as illustrated in Figs. S3(B) c and (C) c.

1. **Measurement sequence**


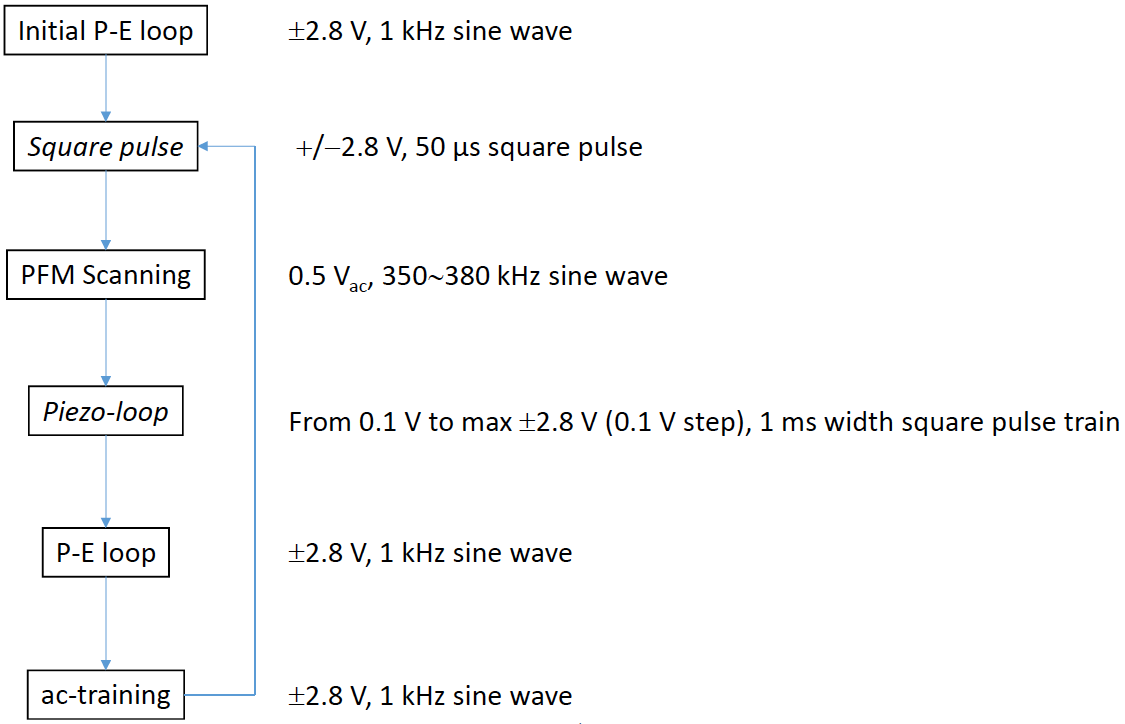


**(B) – Capacitor L (poling with a square pulse of + 2.8 V, 50 µs)**

a)


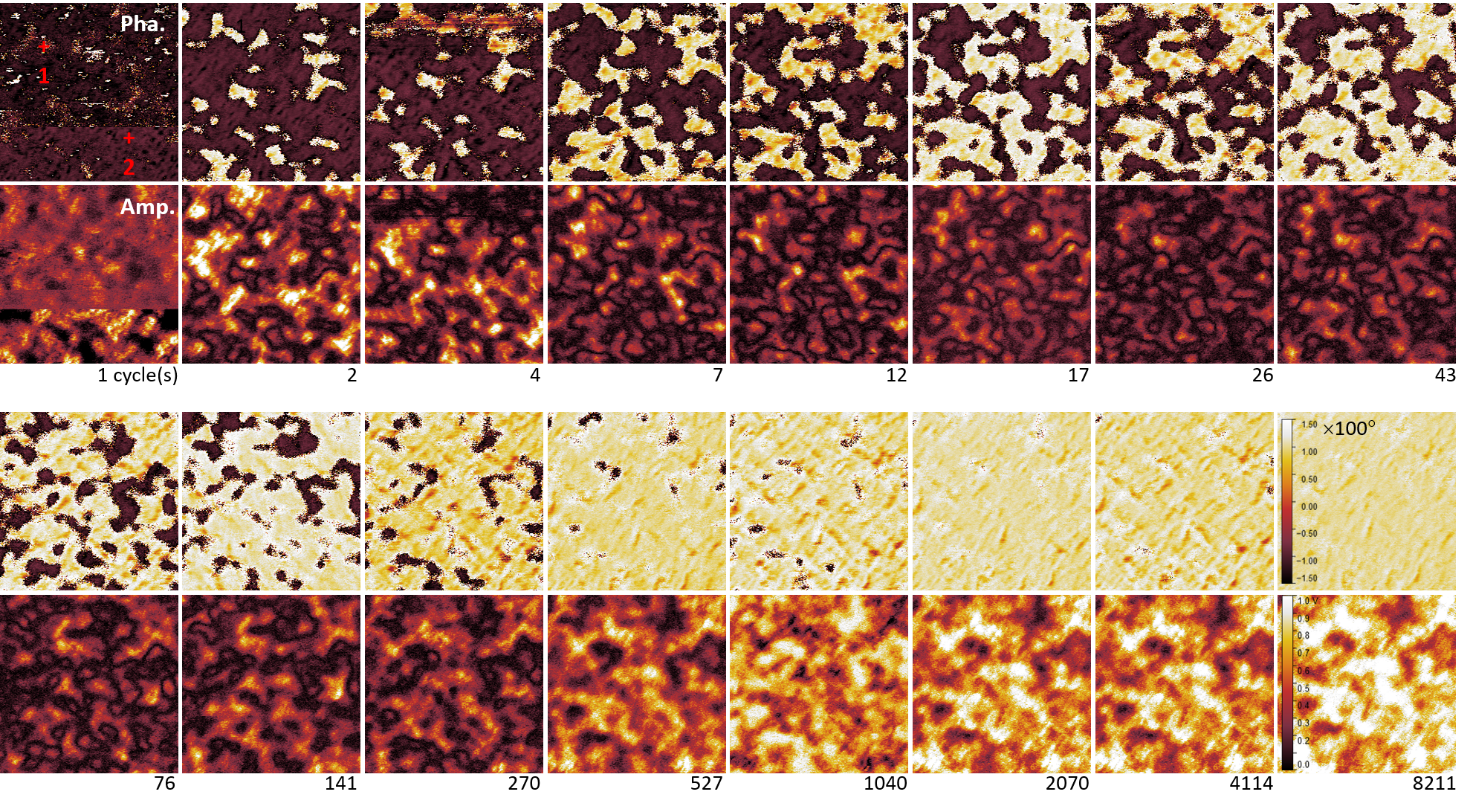


b)


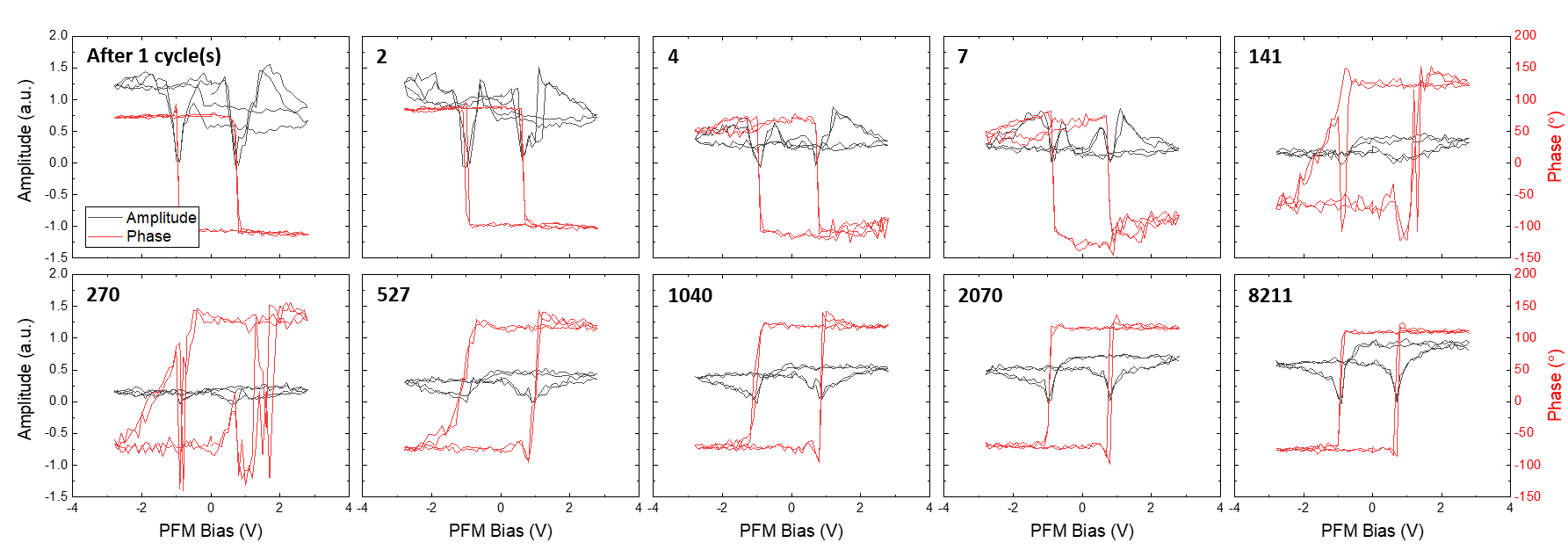


c) d)

**
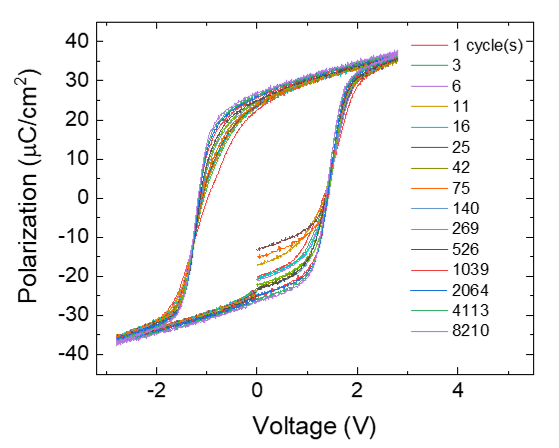

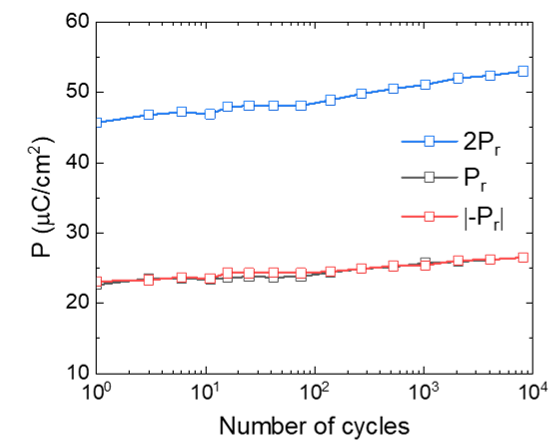
**

**(C) – Capacitor R (poling with a square pulse of - 2.8 V, 50 µs)**

a)


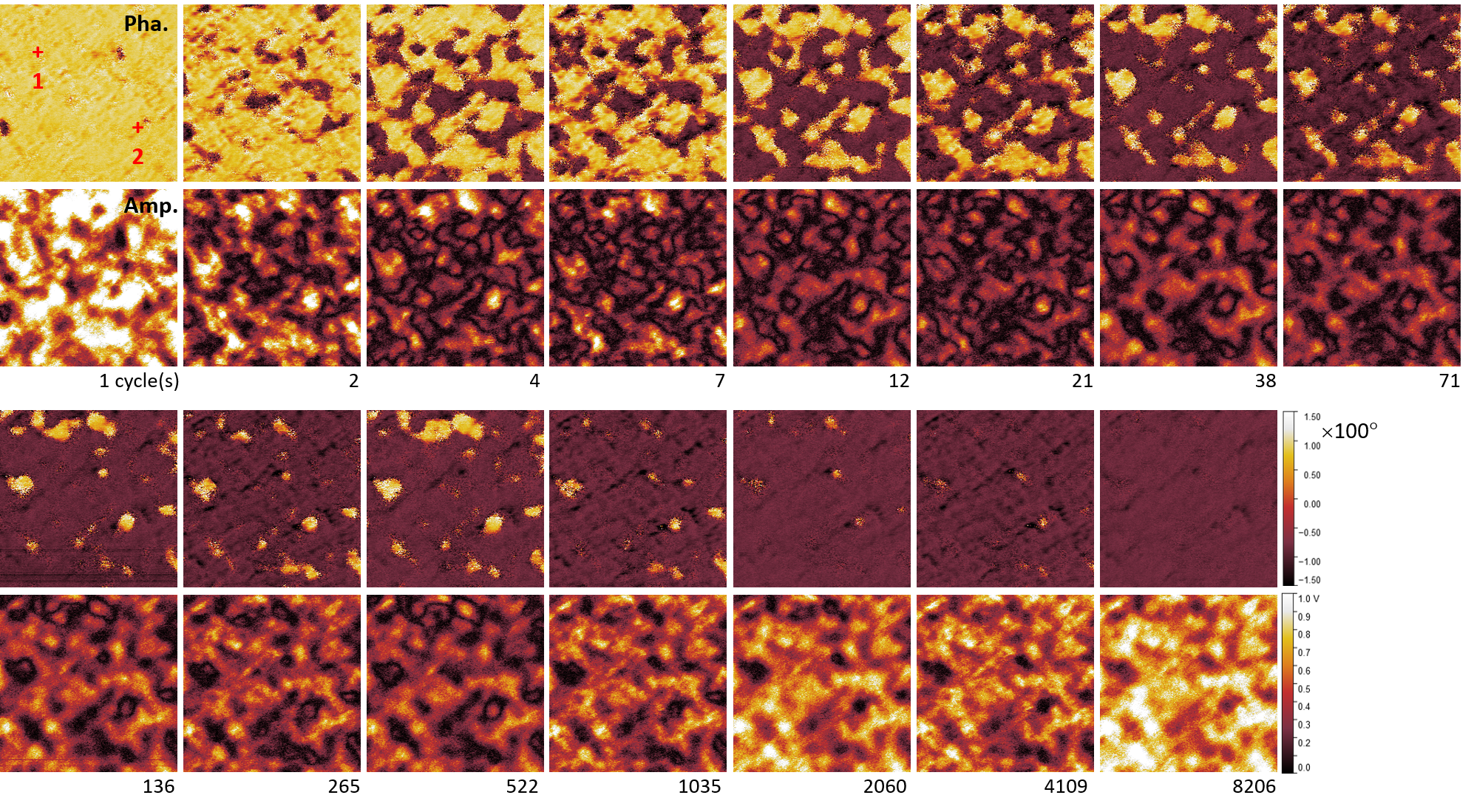


b)


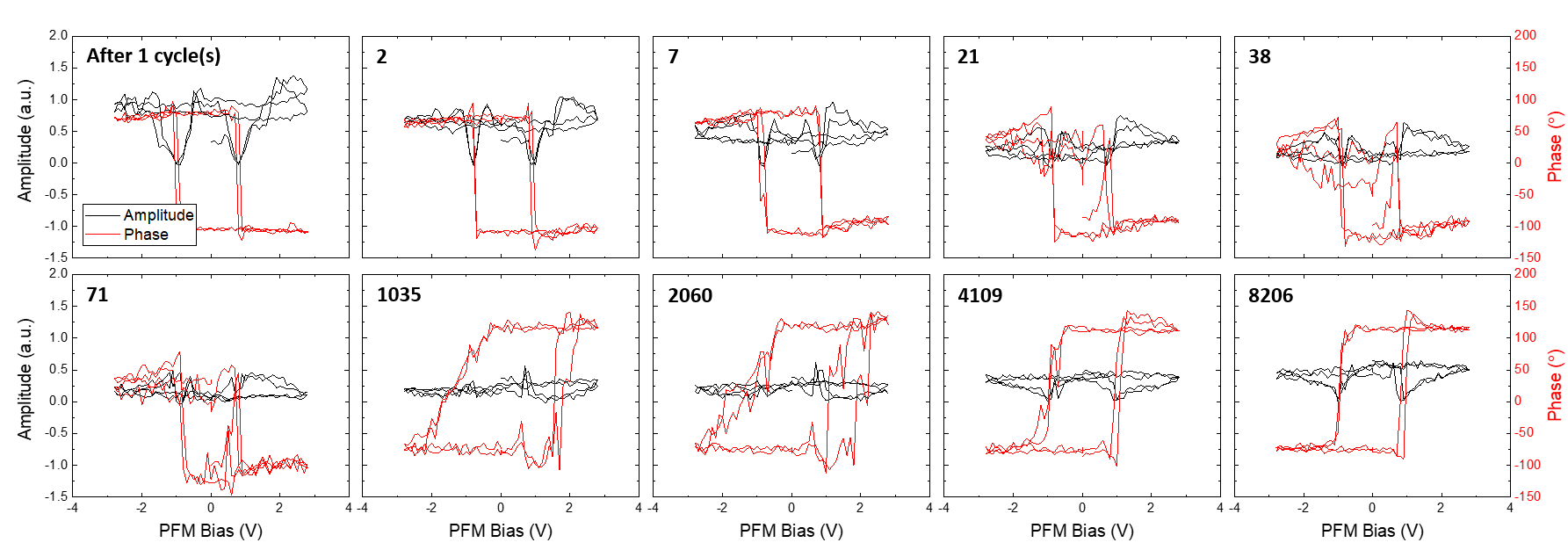


c) d)


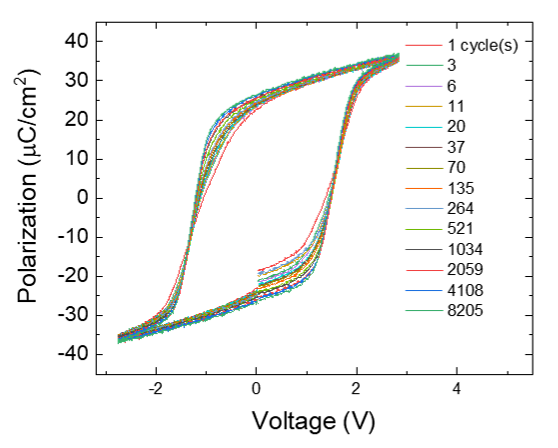

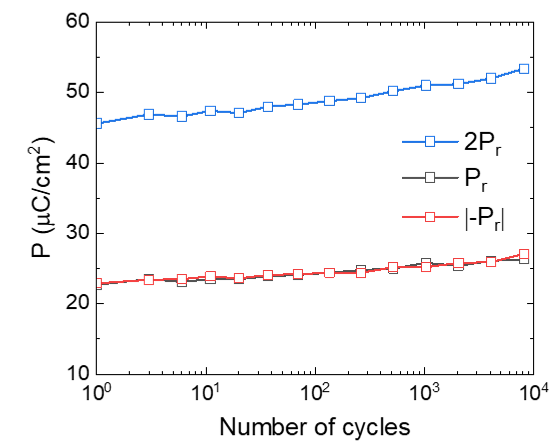


**Fig. S3:** **Measurements on two different W/HZO/W capacitors as a function of the number of ac electrical cycles.** (A) For each capacitor, after an initial P-V loop measurement, the following sequence is repeated: a square pulse is applied for poling the capacitor (+ 2.8 V for capacitor L and - 2.8 V for capacitor R, 50 µs), then a PFM image is recorded on a 1 x1 µm^2^ area (the number of ac cycles including the initial one is indicated below each image – the same area is investigated throughout the whole sequences), followed by local switching spectroscopy (off-field) measurements on 2 different locations (noted 1 and 2 in red on the first PFM phase image), followed by a P-V loop measurement, followed by ac cycles (sine wave waveform, ± 2.8 V, 1 kHz). (B) Capacitor L: a) Evolution of the PFM phase and amplitude images upon increasing number of ac cycles after application of a positive poling square pulse (+ 2.8 V), b) PFM switching spectroscopy (amplitude and phase) measured on location 2 as indicated in red on the first PFM phase image, c) P-V loops at the different stages of ac cycling, and d) corresponding remanent polarization - (C) Capacitor R: a) Evolution of the PFM phase and amplitude images upon increasing number of ac cycles after application of a negative poling square pulse (- 2.8 V), b) PFM switching spectroscopy (amplitude and phase) measured on location 1 as indicated in red on the first PFM phase image, c) P-V loops at the different stages of ac cycling, and d) corresponding remanent polarization

**Fig. S4.** **Bias-on PFM switching spectroscopy**. (a-f) Bias-on PFM switching spectroscopy loops as a function of the applied switching dc bias at a fixed location on the W/HZO/W capacitor during the ac cycling process in the pristine state (a), after 25 cycles of ac cycling (b), after 100 cycles of ac cycling (c), after 500 cycles of ac cycling (d), after 1000 cycles of ac cycling (e), and after 5000 cycles (f) of ac cycling.





**Fig. S5:** **Imprint on two different W/HZO/W capacitors (L and R) as a function of the number of ac electrical cycles.** (a) Coercive voltages V_c_^+^ and V_c_^-^ extracted from the P-V loops shown in (c) of Fig. S3 for the two capacitors named L and R and imprint (calculated as $\frac{(V_{c}^{+}+V_{c}^{-})}{2}$), as a function of ac cycles. The imprint changes by less than 0.1 V over 10 000 cycles.


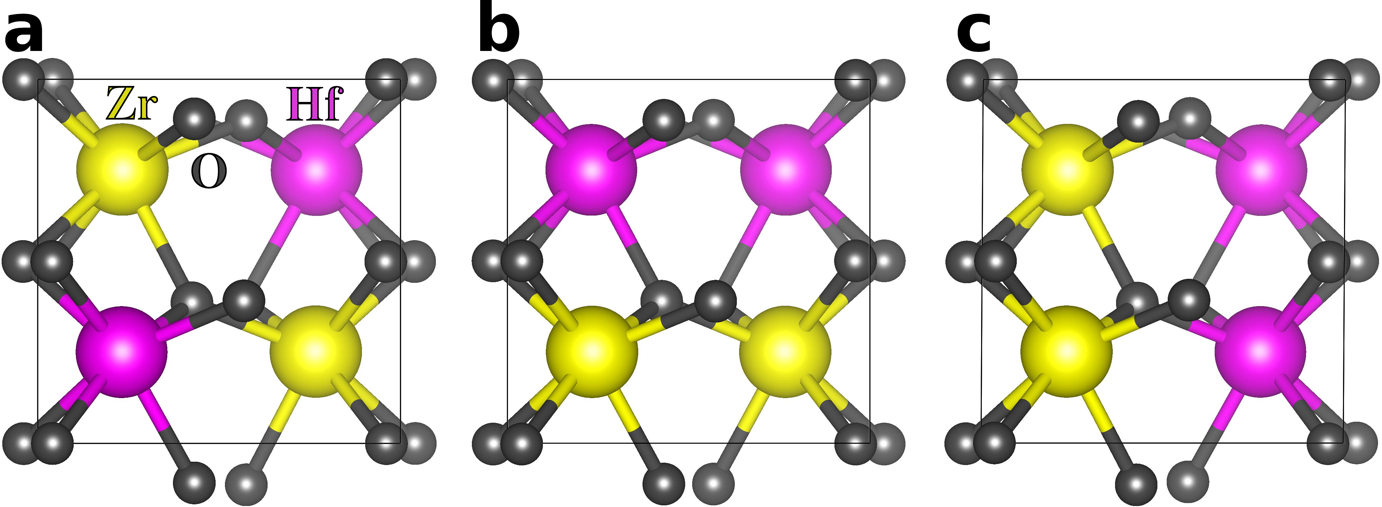


**Fig. S6. Simulated cells. a**. Unit cell employed for the simulations of the o-III phase of HZO. The Hf and Zr cation ordering preserves the 2_1_ screw axis of the pure HfO_2_ ortho-III state. **b** and **c** show other possible Hf/Zr cation orderings which we also considered; the effect of the cation ordering on the computed properties was found to be negligible.


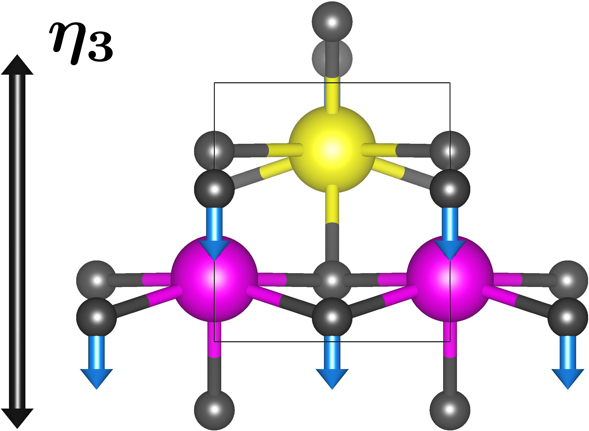


**Fig. S7.** **Sketch illustrating the computed longitudinal piezoresponse of the ortho-IV phase of HZO.** A tensile strain along the polar axis $\eta_{3}>0$ induces a downwards movement of the polar-active oxygen atoms and hence an increase of the polarization, which yields $d_{33}>0$. The atomistic mechanism is thus analogous to the one described by Dutta *et al.* [27] for the ortho-III phase but rendering a positive piezoresponse in this case.
